# Do Land Use Structure Changes Impact Regional Carbon Emissions? A Spatial Econometric Study in Sichuan Basin, China

**DOI:** 10.3390/ijerph192013329

**Published:** 2022-10-15

**Authors:** Heping Li, Tao Lin

**Affiliations:** Faculty of Architecture and Urban Planning, Chongqing University, 400044 Chongqing, China

**Keywords:** carbon emissions, information entropy, land use structure, spatial econometric model, Sichuan basin

## Abstract

Human activities are closely related to carbon emissions and the mechanism of land-use structure change on carbon emissions is unclear. In this study, 143 counties in the Sichuan Basin of China were used as sample units, and the land use structure of each sample unit in the Sichuan Basin was measured by applying the information entropy theory, analyzing the spatial and temporal evolutionary characteristics and the influencing relationships of land use structure and carbon emissions in the Sichuan Basin, by spatial econometric analysis of panel data on carbon emissions and information entropy of land use structure over five time periods from 2000 to 2018. The results indicate that: the carbon emission intensity and information entropy of land use in the Sichuan basin are increasing over the years, and the cross-sectional data reflect inconsistent spatial distribution characteristics, with greater changes around large cities; both carbon emissions and land use structure are spatially auto-correlated, the information entropy of land use positively affects carbon emission intensity; carbon emissions have positive spillover effects, and changes in land use structure have no obvious regional impact on surrounding areas; there may be potential threshold areas for the impact of land-use structure change on carbon emissions. This study has certain reference value for land use planning and carbon emission reduction policies.

## 1. Introduction

Climate change is the greatest non-traditional challenge to human development, and the world is facing the double pressure of economic transformation and carbon reduction. Since the 1990s, the United Nations Framework Convention on Climate Change [1], the Kyoto Protocol [2] and the Paris Agreement [3] have been adopted as three important international legal documents to address climate change, laying down the legal basis and framework for greenhouse gas emission reduction in the world. On 22 September 2020, China announced at the 75th session of the United Nations General Assembly that it would achieve carbon peaking by 2030 and carbon neutrality by 2060. The year 2021 was the year of full implementation of the Paris Agreement, and around the 26th Conference of the Parties to the United Nations Framework Convention on Climate Change in November 2021, many countries proposed new emission reduction targets. During the conference, China and the U.S. released the Declaration for Enhanced Climate Action in the 2020s, setting forth their respective plans for carbon emissions over the next decade. At present, China’s carbon emission intensity is still on the rise and China faces serious challenges in achieving the emission reduction targets set for 2030 and 2060.

The land is the spatial carrier of human production and life, the carbon reduction scheme based on land use structure is a common concern of the global academic community. Land use and land cover change (LUCC) have obvious carbon effects, and the carbon emission effects of different land use types (construction land as a carbon source; forest land and grassland as a carbon sink) have been extensively studied [4,5,6]. Ghaffar Ali and Nitivattananon (2012) argue that the decrease in industrial production and forestry land is responsible for the increase in carbon emissions in Pakistan [7]; Alejandro Carpio et al. (2021) analyzed the effects of urban land growth and land class change on carbon emissions in the Monterrey Metropolitan area, Mexico [8]; Rio Aryapratama and Stefan Pauliuk (2022) estimated the life-cycle carbon emissions of wood products in Indonesia for different land conversion types over time [9]; Asif Raihan and Almagul Tuspekova (2022) analyzed the relationship between agricultural land and carbon emissions, by the Dynamic Ordinary Least Squares (DOLS) method, in Peru [10]. Land use structure is an indicator of economic development and carbon emissions; there is a correlation between land use efficiency and carbon emissions [11,12,13,14,15,16]. Information entropy is used to measure the disorder of information, first proposed by the American mathematician Shannon in 1948 [17]; the concept of information entropy has been introduced to many fields. Information entropy was introduced as a unit of measurement to characterize the change of land use structure, there have been numerous studies on the change of land use structure in multiple regions and at multiple scales [18,19,20]. For example, the review of Boltzmann entropy in the application to land use and landscape patterns by Gao, P., and Li, Z. [21]; Yang Chao et al. explored the structure relationship of land use in Guangdong Province by using information entropy of land use [22]; Gülçin, D. used information entropy correlation theory to assess the correlation between ecological connectivity and ecological environment [23]; and Xu Ze et al. introduced the information entropy theory to assess the decoupling relationship between land use structure and carbon emissions [24]. The spatial econometric analysis model makes up for the shortcomings of classical econometric methods on the spatial relationship of elements, while overcoming the lack of quantitative analysis based on GIS platform for long-series data processing. For example, Bu, Y. et al. analyzed the impact of population migration on energy consumption and carbon emissions in China using the Spatial Durbin Model [25]; Xiong Chang-sheng et al. used the spatial econometric model to analyze the impact of provincial infrastructure inputs on socio-ecological system vulnerability in China [26]; Wang X. et al. analyzed the impact of digital finance on carbon emissions in Chinese prefecture-level cities using a spatial econometric approach [27]; Coetzee C.E. and Kleynhans, E.P. analyzed the correlation between night-time lights and GDP in South Africa using the spatial econometric model [28]; Salvo, M.D. et al. used spatial econometric methods to evaluate the impact of climate change on the performance of wine companies in Moldavia, Romania [29].

The above studies provide a solid theoretical basis for the study of carbon emissions and land use structure, but fewer studies analyze carbon emissions econometrically by quantifying the land use structure, and most of them focus on cross-sectional data analysis, lacking the analysis of the lag and spillover effects of land use change with carbon emissions, so the evolutionary mechanisms of land use change and carbon emissions still need to be further explored. The key to exploring the relationship between land use structural changes on carbon emissions is to transform land use structure into a quantifiable and observable parameter, and then conduct spatial econometric analysis. As mentioned earlier, Shannon’s information entropy theory, which was introduced to the field of land use research by many researchers, is used to characterize crucial characteristics of the land use structure change. This study takes land use structure and carbon emissions as the research object; takes a relatively complete geographical unit, the Sichuan basin of China, as a sample unit with county administrative regions; and uses the spatial econometric analysis model to study the evolutionary trends between land use structure and carbon emissions, based on five periods of panel data from 2000 to 2018, which complements the research results on the relationship between environmental factors on carbon emissions.

## 2. Material and Methods

### 2.1. Study Area

**Figure 1:**
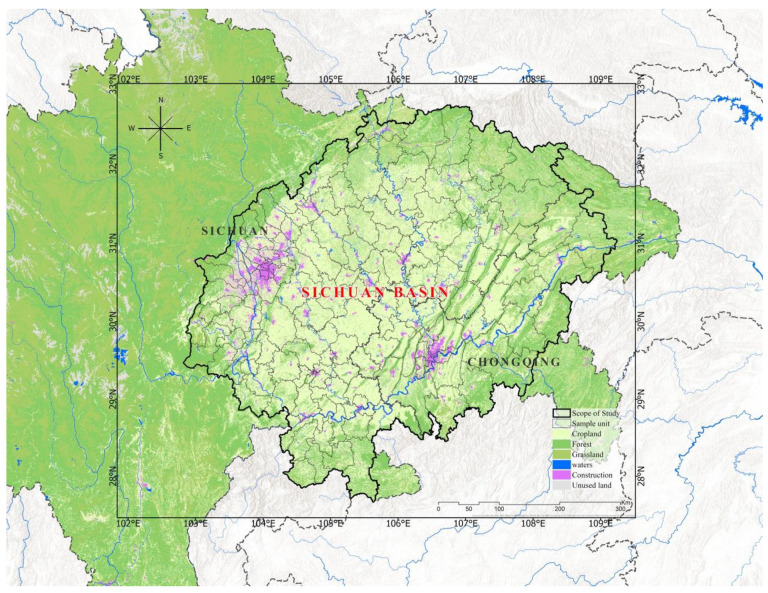
The study area of Sichuan Basin: Land use in 2015.

The study area is the Sichuan Basin, one of the four major basins of China, located in southwestern China (106° E−110° E, 29° N−31° N), surrounded by the Tibetan Plateau, the Daba Mountains, the Huaying Mountains, and the Yunnan-Guizhou Plateau, between 160 and 3000 m above sea level. As shown in Figure 1, the study area is one of the most populated regions in China, a complete geographic unit, with a large proportion of forested and cultivated land. The Sichuan basin’s terrain is varied, with mountainous, hilly, and plain environments, the region has undergone fast urbanization, considerable changes in land use patterns, and uneven economic development over the last 20 years, particularly in Chengdu and Chongqing, where the “Chengdu-Chongqing” region has been the focus of China’s future development in recent years. As a result, it is critical to investigate the relationship between land use and carbon emissions in the Sichuan basin in order to develop land use regulations and meet future carbon emission reduction targets. The study area contains 143 counties in two regions of Sichuan province and Chongqing city, with a total area of about 273,717 Km^2^. This study takes 143 counties in the Sichuan basin as samples to analyze the changing relationship between land use structure and carbon emissions.

### 2.2. Data Sources

This study contains five study periods, 2000, 2005, 2010, 2015, and 2018, where the last period of carbon emissions is 2017 data, and the data used include statistical data, vector data, and raster data. The list of data is shown in Table 1.

The statistics are carbon emissions, obtained from China Carbon Emission Accounts and Datasets [30], a database jointly established by several Chinese and foreign research institutions, which is the longest time span and widest coverage of carbon emission data of county-level administrative regions in China, spanning from 1997 to 2017, and can provide carbon emission data support for this study. A total of 5 periods of carbon emission data from 2000/2005/2010/2015/2017 were selected as the basic data of this study.

Considering that carbon emission intensity may be affected by many factors such as population, economy, and consumption, this study involved these three factors as control variables in the econometric analysis, where Gross Domestic Product data are used for the economy and the Total Retail Sales of Consumer Goods data are used for consumption, which were obtained from the statistical yearbooks of Sichuan Province and Chongqing Municipality over five study periods.

The raster data are land use data from the China’s Multi-Period Land Use Land Cover Remote Sensing Monitoring Dataset (CNLUCC) released by the Chinese Academy of Sciences [31], which is based on the Landsat remote sensing image data from the United States and includes five periods of land use data in 2000/2005/2010/2015/2018 with a resolution of 30 m. This dataset divides the data into 6 categories: cropland, forest, grassland, waters, construction, and unused land. In particular, this study mainly responds to the relationship between land use change and carbon emission effects. Since the last period of carbon emissions was 2017 and the last period of land-use cover data was 2018, the comparison revealed that the land cover and carbon emissions did not change much during one year, and the 2017 carbon emissions data and 2018 land use data were included in the econometric analysis in order to ensure the sample size and the timeliness of the study.

The county-level administrative districts data obtained from the Resource and Environmental Science and Data Center [32], Institute of Geographical Sciences and Resources, and the Chinese Academy of Sciences (RESDC). The study area spatio-temporally spans 15 years, and this study will combine the change of administrative division with carbon emission data during the study period for consistency correction. The study area contains a total of 143 counties.

### 2.3. Method

**Figure 2:**
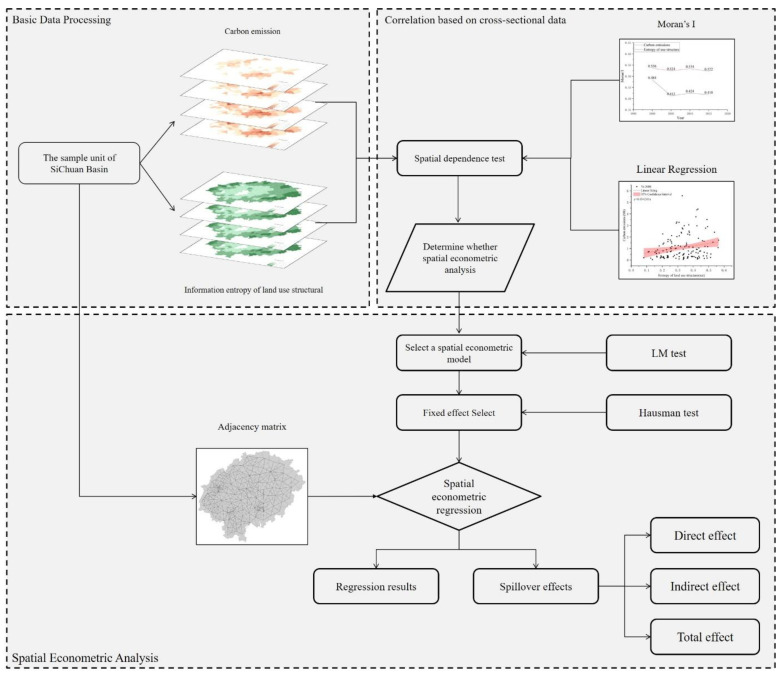
Research framework.

This paper takes the Sichuan Basin in China as the research area and 143 counties as the sample unit to formulate the research framework (Figure 2). ① GIS was used as a platform to calculate the information entropy of land use structure for each unit using land use data, 5 periods from 2000 to 2018. The carbon emission data were entered into 143 sample units; ② Spatial correlation analysis was conducted for carbon emissions (dependent variable C) and information entropy of land use structure (independent variable E), respectively, for the study area by Moran’s I, linear regression analysis to determine whether each variable has autocorrelation; ③ Calculation of the adjacency matrix w of sample cells using GeoDa to participate in spatial panel analysis; ④ The Stata platform, LM test, Hausman test, LR test, and Wald test were used, to determine whether SDM can degenerate into SLM and SEM models, and to compare and select the optimal fixed effects; ⑤ Using spatial econometric regression, we examined the impact of land-use structure information entropy on carbon emissions and spillover effects, while also using population, economic, and consumption data as control variables.

#### 2.3.1. Sample Unit

The sample unit of this study is 143 county-level administrative regions within the Sichuan basin, and the carbon emission data of five periods from 2000 to 2018; the land-use mixture data calculated in this study are joined into the 143 county-level administrative regions as the sample unit of this study for spatial econometric analysis.

#### 2.3.2. Land Use Structure Information Entropy Measurement

The theory of information entropy was proposed by the American mathematician Shannon in 1948, based on the theory of dissipative structure, to deduce the law of change of entropy and determine the direction of system evolution, by measuring the degree of disorder of information. Information entropy can be used to reflect the characteristics of land use structure, and many scholars have used information entropy to characterize the degree of order and evolution of land use structure [33,34,35]. Referring to the existing research results, the formula of information entropy of land use structure is as follows:

(1) $$E=-\overset{n}{\underset{i=1}{\sum }}\;\left[\left(bi/m\right)\log\left(bi/m\right)\right]$$

where E denotes the information entropy of land use structure (nat, measurement unit of information entropy), and the level of information entropy reflects the equilibrium degree of land use; *n* is the number of land use types; *bi* is the area of the *i*-th land use type in a county unit; *m* denotes the area of all land use types in a county unit.

#### 2.3.3. Spatial Autocorrelation Model

This study uses the global spatial autocorrelation test to analyze the spatial correlation and the degree of variation of the region as a whole [36], which is a measure of spatial autocorrelation proposed by Moran, P.A. (1950) to describe the degree of spatial agglomeration or dispersion of things or phenomena, and the spatial autocorrelation test is a prerequisite for spatial econometric model analysis [37]. In this study, Moran’s I was used to test the autocorrelation of the factor, and the spatial weight matrix is the Queen’s adjacency matrix. The formula of global Moran’s I formula is as follows:

(2) $$I=\frac{\sum_{i=1}^{n}\sum_{j=1}^{n}w_{ij}\left(x_{i}-\bar{x}\right)\left(x_{j}-\bar{x}\right)}{S^{2}\sum_{i=1}^{n}\sum_{j=1}^{n}w_{ij}}$$

where I is the Moran’s I reflecting the strength of global spatial autocorrelation, and its value is in [−1, 1], the more the value tends to 1, the stronger the spatial positive correlation, the more it tends to −1, the stronger the spatial negative correlation, and zero indicates a spatial random distribution; *x_i_*, *x_j_* are the observed values of sample units *i* and *j*, respectively; $\bar{x}$ is the sample mean; *S^2^* is the sample variance. *n* is the number of samples. *w_ij_* is the spatial weight, this study uses the Queen adjacency matrix. *w_ij_* is 1 if sample unit *i* and sample unit *j* are adjacent to each other, and 0 otherwise.

#### 2.3.4. Spatial Econometric Model

*Everything is related to everything else, but near things are more related than distant things*, which is the first law of geography [38]. Carbon emissions and land-use structure change are continuous processes, which not only show the spatial characteristics of clustering and spillover in cross-sectional time, but also have lagging effects from the past to the future in the time dimension. Cliff A.D et al. (1973), Anselin (1988), Arbia (2006), and other scholars developed spatial econometrics and established spatial econometric models to incorporate the spatial interaction of economic activities and spatial structure issues into the scope of spatial econometrics [39,40].

This study uses a spatial econometric model to analyze the impact of land use change on carbon emission intensity [39,41,42,43]. The spatial econometric model contains three basic models: Spatial Lag Model (SLM); Spatial Error Model (SEM); and Spatial Durbin Model (SDM). The SDM is a special case of the Spatial Lag Model (SLM) and Spatial Error Model (SEM).

The formula of the Spatial Lag Model (SLM) formula is as follows:

(3) y=ρWy+Xβ+ε

The formula of the Spatial Error Model (SEM) formula is as follows:

(4) $$y=X\beta +u,u=\rho \mathrm{Wy}+\varepsilon ,\varepsilon \sim N\left(0,\delta^{2}I_{n}\right)$$

The formula of the Spatial Durbin Model (SDM) formula is as follows:

(5) y=ρWy+Xβ+WXδ+ε

where y is the dependent variable, representing carbon emissions; ρ is the autoregressive coefficient; X is the matrix of independent variable, representing the information entropy of land use structure, *β* is the correlation coefficient; W is the spatial weight matrix, and *δ* is the corresponding coefficient vector; *ε* is the stochastic disturbance.

#### 2.3.5. Variables

Carbon emissions intensity is the dependent variable (C), and land use information entropy is the independent variable (E). Considering that carbon emissions may be influenced by other variables, this study adds total regional Population (POP), Gross Domestic Product (GDP), and Total Retail Sales of Consumer Goods (RSC) as control variables, logarithmically transformed, for econometric analysis (Table 2).

## 3. Results

### 3.1. Temporal and Spatial Changes

#### 3.1.1. Carbon Emission

**Figure 3:**
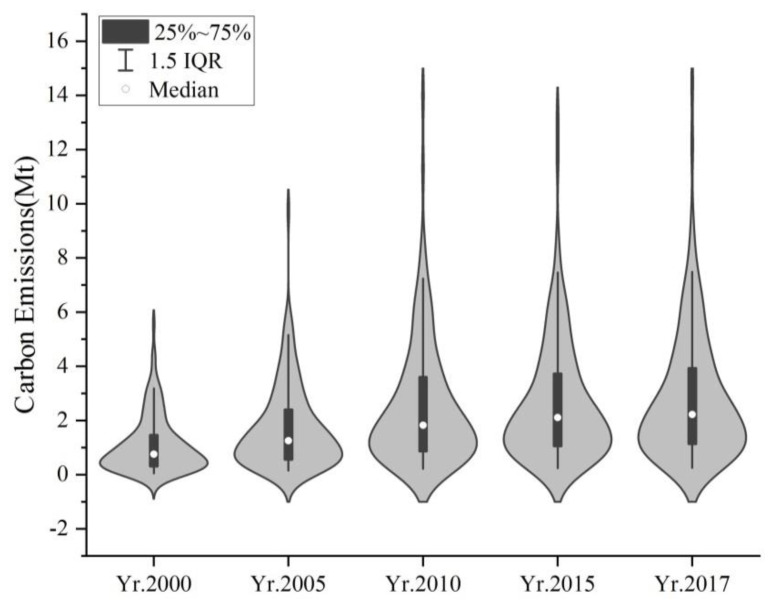
Carbon emission Violin Plot: 2000−2017.

**Figure 4:**
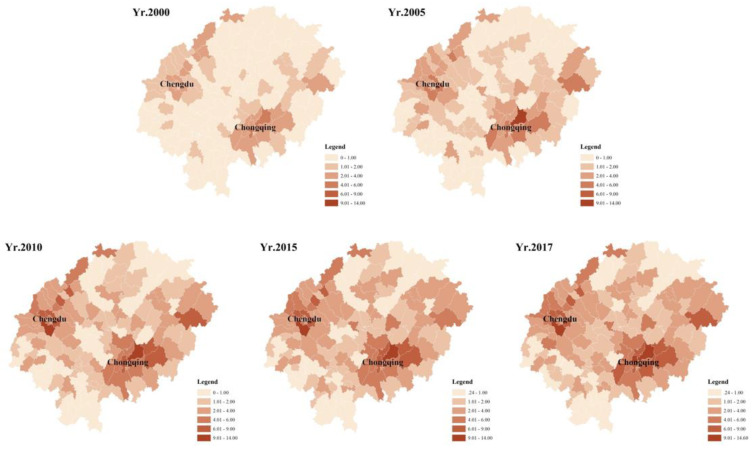
Carbon emissions distribution map: 2000−2017.

Analyzing the carbon emissions of 143 sample units in the Sichuan Basin from 2000 to 2017, it can be concluded: ① The total carbon emissions in the Sichuan Basin grew from 156.20 Mt (million tons) in 2000 to 402.89 Mt in 2017, with average annual growth rate of 5.73%, of which the median carbon emissions in 2000 was 0.76 Mt and the median carbon emissions in 2015 was 2.28 Mt. The carbon emissions intensity in the Sichuan Basin is a trend of faster growth (Figure 3); ② The distribution of carbon emission intensity from each period is shown on the graph (Figure 4).The carbon emission intensity of each region within the Sichuan basin shows a more significant agglomeration. The carbon emission intensity in the eastern region of the basin is higher than that in the western region, and the carbon emission intensity distributed in the counties under the jurisdiction of Chongqing and Chengdu is significantly higher; ③ The differences in the increase in carbon emission intensity among the sample units are large, with 143 sample units increasing between 0.17 Mt and 8.94 Mt. Among them, the carbon emission intensity of the counties under Chengdu and Chongqing is higher; the carbon emissions in the south and north of the Sichuan Basin are relatively low.

Land use change is the result of the combined effect of natural conditions and socio-economic development. Changes in land use structure in time and space are closely related to changes in carbon emission intensity, and we use information entropy as a measure of land use structure to further explore the correlation between changes in land use structure and changes in carbon emission intensity.

#### 3.1.2. Information Entropy of Land Use Structure

**Figure 5:**
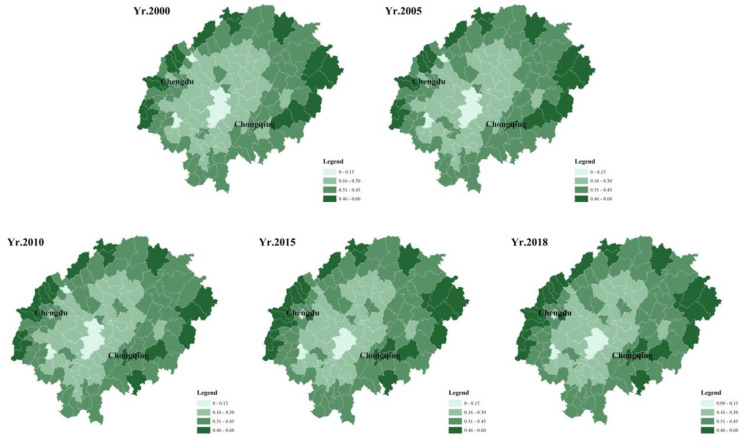
Information entropy of land use structure distribution map: 2000–2018.

Based on the formula (1), this study calculated the land use structure information entropy of 143 sample units within the Sichuan basin. Relating the information entropy to the 143 sample units, the spatial distribution of the information entropy of land use structure in the study area for five periods from 2000 to 2018 was obtained (Figure 5).

The following phenomena were obtained: ① The land use structure information entropy of each sample unit within the basin showed a large increase, and the average information entropy of 143 sample units in the Sichuan basin increased from 0.33 nat to 0.36 nat, with an increase of 0.03 nat. From the average, the overall land use structure, in general, did not seem to change much in 18 years’ time; ② As with the change in carbon emissions, the Yubei district of Chongqing is the region with the largest change in information entropy, with a difference of 0.14 nat, and the Pidu district of Chengdu is 0.11 nat; the Wuhou district of Chengdu shows a significant decrease in information entropy of −0.24 nat and an increase in carbon emissions of 2.06 Mt.; ③ There are differences in the distribution pattern of carbon emission intensity; the sample units with higher carbon emission intensity are distributed around the big cities such as Chongqing and Chengdu, while the areas with higher information entropy of land use structure are distributed around the basin, and the information entropy of the central region of the basin is lower than that of the peripheral regions, indicating that the land use structure is more complex at the edge of the basin; ④ Within the Sichuan basin, not all sample units have increased information entropy of land structure, and there is a large heterogeneity of land use structure between regions, with 15 sample units having negative information entropy of land use structure, the lowest being Wuhou district with information entropy of 0.34 nat in 2000, 0.27 nat in 2005, 0.17 nat in 2010, 0.10 nat, and 0.10 nat in 2018.

#### 3.1.3. Correlation Based on Cross-Sectional Data

**Figure 6:**
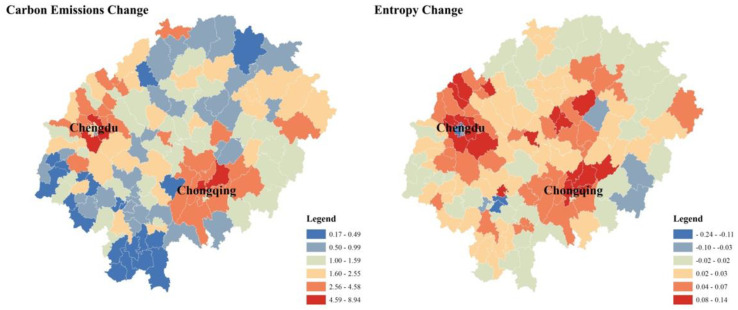
The 2000−2017 carbon emissions change and 2000−2018 entropy change (Natural Breaks, Jenks).

As noted above, we found that in the single-period cross-sectional data of carbon emissions and land use structure information entropy, the spatial distribution of carbon emissions was not consistent with the spatial distribution characteristics of information entropy, which does not seem to support the original hypothesis that land-use structure change affects carbon emission intensity. However, when we make a distribution map of the difference between 2000 and 2018 (Figure 6), we intuitively find that Regions with a large increase in the information entropy of land-use structure information are highly consistent with regions with a large increase in carbon emission changes.

**Figure 7:**
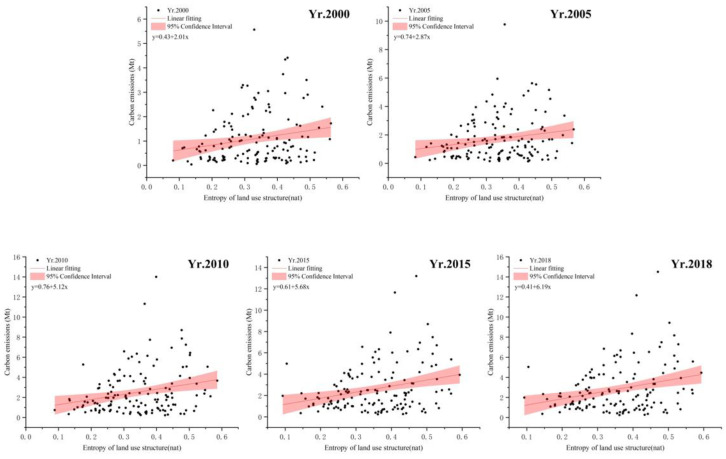
The 2000−2018 entropy with carbon emissions Scatter Plot.

Using the information entropy of each period as the independent variable and carbon emission as the dependent variable, a linear regression was conducted to draw a scatter plot (Figure 7), and the results showed that information entropy and carbon emission intensity were positively correlated, among which, the regression coefficient was 2.01 in 2000 and 6.19 in 2018, which indicated that the degree of influence of land-use structure change on carbon emission was becoming stronger.

After the above analysis, we tentatively conjectured that the static land-use structure information entropy-distribution pattern had little correlation with the spatial distribution of carbon emission intensity, that a linear regression analysis of the data at the cross-sectional level can only indicate a possible correlation between land use structural changes and carbon emissions, and that in order to further explore the influence relationship, spillover effect, and lag effect between them, we would use the spatial econometric model to estimate the carbon emission intensity and information entropy of land use structure.

### 3.2. Spatial Econometric Analysis

**Figure 8:**
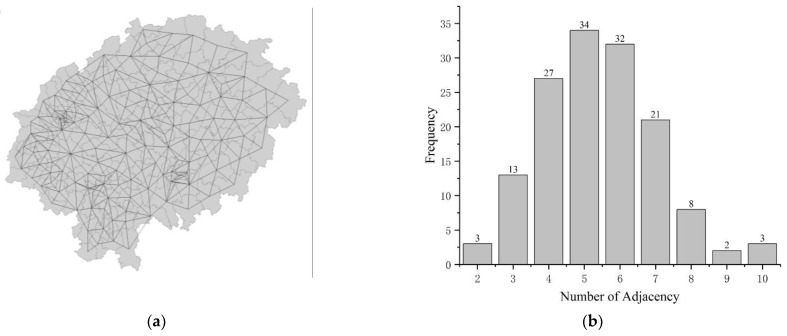
The adjacency matrix of the sample unit. (**a**) The connected graph of the adjacency matrix. (**b**) Neighborhood statistics for each sample unit.

According to the description of the first law of geography [38], geographic attributes are interrelated in spatial distribution and have the characteristics of Clustering, Random, and Regularity distribution. According to the process of this paper (Figure 2), the GeoDa platform is used to generate the 143 × 143 adjacency matrix W (Figure 8); the carbon emission and land-use structure information entropy of the four periods are tested for Moran’s I autocorrelation, and if there is an autocorrelation feature, further spatial analysis was conducted.

#### 3.2.1. Spatial Autocorrelation Test

**Figure 9:**
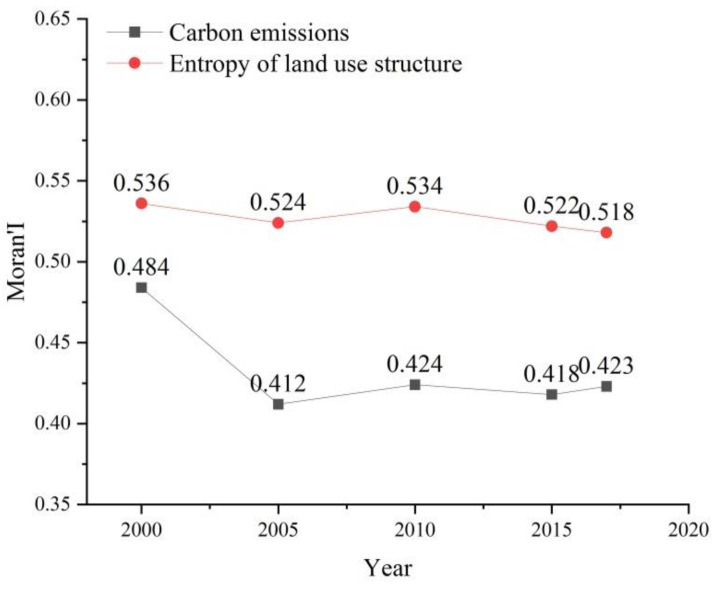
Moran’s I of carbon emissions and information entropy of land use structure: 2000−2018.

**Figure 10:**
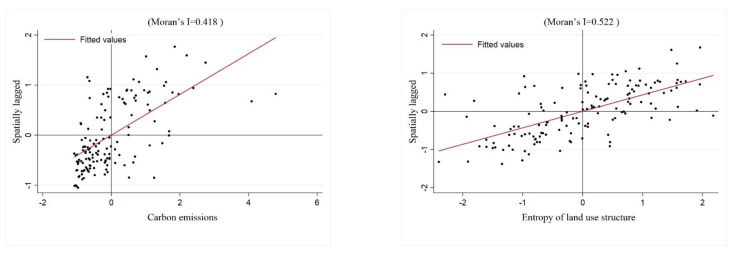
Moran’s I scatter diagram of carbon emissions and information entropy of land use structure in 2015.

Using the 143 × 143 adjacency matrix W, the Moran’s I indices of carbon emission intensity and information entropy of land use structure are calculated according to Equation (2), and the Moran’s I indices of carbon emission and information entropy of land use structure in the Sichuan basin are in the range of 0.412–0.484. The Moran’s I indices of land use structure information entropy are in the range of 0.518–0.536, respectively, during the period of 2000–2018, both of which pass the 5% significance test (Figure 9). In addition, from the Moran’s I scatter plot of carbon emissions and information entropy of land use structure in 2015 (Figure 10) it can be seen that the scatter points are mostly distributed in quadrant 1 and quadrant 3, indicating that both carbon emission intensity and land use structure have spatial autocorrelation characteristics, and there are aggregation distribution characteristics, which can be analyzed by using the spatial econometric model in this study.

The spatial clustering of carbon emissions in the Sichuan basin may be due to two reasons: (i) the geographical development has the characteristics of a growth pole nucleus, that is, the rapid development of a certain point will drive the development of adjacent surrounding areas; (ii) the unique financial decentralization and local political system in China, which prompt localities to increase their input in some areas, and so the intensity of carbon emissions emission is relatively concentrated. The spatial clustering of the information entropy may also be due to two reasons: (i) administrative districts within the same geographical unit have similar land-use patterns; (ii) neighboring sample units have similar policies and anthropogenic activities in response to conditions such as construction land and natural resources.

#### 3.2.2. Model Selection

In this study, spatial econometric analysis was performed in the Stata platform. The panel data of land-use structure information entropy and carbon emissions in five periods were analyzed: ① Lagrange Multiplier (LM) test, the *p*-value of both the SEM model and SLM model were significant at 1% level, and the SDM model combining both could be selected in this study; ② Hausman test, the results passed the 1% significance level test, and the fixed effect model was better than the random effect model in this study; ③ Likelihood ratio (LR) test, the original hypothesis of degrading the SDM model into SLM and SEM model did not accept the original hypothesis that the SDM model degenerates into SLM, SEM model; ④ Wald test, did not support the degeneration of SDM into SLM, SEM model. This study adopts the SDM model for spatial econometric analysis.

From the test results, carbon emission intensity, which is influenced by the spatial lag of the dependent variable, is also influenced by the spatial lag of the independent variable; further SDM regression analysis is conducted to test three models of spatial fixed effect (sFE), temporal fixed effect (tFE), and spatio-temporal fixed effect (stEF), and the results show that the spatio-temporal fixed effect is chosen as superior using the SDM model. In summary, the spatial econometric model of this study is the SDM model with a spatio-temporal fixed effect, and the SDM spatial econometric analysis is conducted with the information entropy of land use structure as the independent variable and carbon emissions as the dependent variable.

#### 3.2.3. Main Estimation Results

(1) Regression results

According to Equation (5), the results of the SDM regression were calculated by importing a 143 × 143 adjacency matrix W with the information entropy of land use structure as the independent variable E and carbon emissions as the dependent variable C, and POP, GDP, and RSC as control variables. The spatio-temporal fixed effect (stFE) was selected for interpretation (Table 3).

The SDM model regression results: ① the main term shows that the regression coefficient of independent variable E is 8.20 and the *p* value is 0.000, which is significant at 1% level, indicating that the increase in land use structure information entropy has a positive effect on carbon emission intensity; ② the Wx term reflects the spatial transmission effect, and the *p* value is 0.064, which accepts the original hypothesis, indicating that the land-use structure information entropy of each county in the Sichuan basin itself does not affect other regions; ③ in the Spatial term, rho responds to the spatial spillover effect of the dependent variable (carbon emission intensity) on the surrounding regions, and the *p* value of the spatial autoregressive coefficient of the Spatial term in the model is 0.000, which is significant at the 1% level, and its coefficient is 0.53, which is positive, indicating that the explanatory variable C has a positive spatial spillover effect on itself;

(2) Spillover effects

The spillover effect decomposition table is obtained through SDM model regression (Table 4), and the following conclusions can be obtained: (i) Direct effect refers to the degree of influence of the information entropy of the land use structure of the explanatory variable in the region on the carbon emissions of the explanatory variable in the region, the direct effect coefficient is 8.35, and the *p*-value is 0.000, the independent variable E is very significant in the direct effect; (ii) the indirect effect refers to the degree of influence of one unit change in the information entropy of land use structure in the surrounding areas on the carbon emissions of the explanatory variable, and the regression results show that the *p*-value is 0.570. This indicates that the change in land use structure of the surrounding sample units have no effect on changes in carbon emissions; (iii) the total effect is the degree of influence of one unit change in the information entropy of land use structure in all regions on the dependent variable C one unit, the degree of influence on the dependent variable C in this region, and the regression results show that the *p*-value is 0.011 and the coefficient is 10.38, indicating that one unit change in the information entropy of land use structure of all regions will bring 10.38 units of influence on carbon emissions.

## 4. Discussion

Taking the Sichuan Basin of China as the study area, 143 counties in the study area were used as sample units, citing the CEADs database carbon emission intensity data, Based on the information entropy theory, the land-use structure information entropy of each sample unit was measured for five periods from 2000 to 2018, the spatial and temporal trends of carbon emissions intensity and land use structure in the Sichuan basin were analyzed, and the correlation effects and spillover effects of carbon emission intensity on the land-use structure information entropy were calculated using the spatial econometric model. The analysis results suggest that carbon emissions in all parts of the study area keep showing an increasing trend, with an average annual growth rate of roughly 5.73%. The information entropy of most sample units is growing, whereas individual sections are dropping, and the SDM regression analysis of 143 sample units in the study area reveals that changes in information entropy have a positive impact on carbon emission intensity.

The increase in information entropy of land use structure reflects that the types and proportions of land use become more complex within a certain geographical space, and the findings of this study show that a more chaotic land-use structure leads to an increase in carbon emissions. Some researchers are concerned that the decrease in forest land will weaken the carbon sink capacity of the environment, and the expansion of construction land will lead to the increase in carbon sources [44,45]. Meanwhile, Liu’s (2016) research demonstrates that soil change influences the carbon sink capacity [46], and the Sichuan basin has experienced rapid urbanization and quick changes in land use structure in the last 20 years, indicating the trend of rapid urban expansion and hence increased carbon emissions. Furthermore, the more complex the land use function, the more carbon emissions intensify, because the land use function is complex, and human travel, work, and activities will become more diverse, inevitably leading to increased carbon emissions [47]; we believe that this conclusion also supports the view of this study, because the larger scale land-use structure change, essentially, also reflects the results of human activities and land interdependence.

We discovered in our study that there are specific units where the land-use structure information entropy rose but carbon emissions declined. Because changes in land use structure and carbon emission intensity are influenced by numerous factors, we cannot rule out the possibility that there are other more relevant reasons in these places causing them to have a negative association, or is it simply a statistical error caused by data accuracy? According to some studies, China’s industrial carbon emissions have an inverted U-shaped non-linear relationship with the level of economic development [48], and it is widely assumed that economic development is consistent with the growth of carbon emissions, however, the backward stage of economic development causes more carbon emissions due to resource underutilization. Therefore, we speculate that there may be certain threshold intervals, and when the information entropy of land use structure is within these intervals, the information entropy shows a negative correlation with carbon emission intensity. To further verify this conjecture, we will also collect more evidence and conduct a more in-depth study in the future.

There may be some shortcomings in this study, for example, due to the lack of more extensive data, this paper only used data from 2000 to 2018. This leads to the inevitable omission of details in the research results, so we will collect carbon emission data with updated years, add more extensive control variables (e.g., policy factors), and explore the relationship between the impact of land-use outcome changes and carbon emissions. In addition, sample units are identified on a larger scale, in regions where information entropy shows a negative correlation with carbon emission intensity, and deeper patterns of land use and carbon emission effects are discovered.

## 5. Conclusions

The study area was the Sichuan basin, and a panel analysis was conducted using carbon emissions and land use data from 2000–2018, with population, GDP, and consumption level as control variables, and using the SDM model. This study shows that the correlation between land use change and carbon emissions’ change exists, and the increase in land-use structure information entropy leads to a greater carbon emission intensity. Due to the type of data and sample size, this study only explores the correlation between land use change and carbon emissions’ intensity change, and fails to deeply reveal the influence of the law of land use change on regional carbon emission intensity, therefore these issues provide directions for future research.

The above findings have some reference significance for controlling carbon emissions in China. Integrating the overall issue of economic development and land-use structure change, it is important to pay attention to the negative impacts of land use change, and when formulating land use policies, consideration should be given to the impact of land use change on carbon emission control. Our research shows that when the land use structure becomes chaotic (the entropy value reflects the degree of mixing), the carbon emission intensity will be greater. Land use planning and policies should be based on the principle of smart and compact, avoiding excessive encroachment on forests and waters, and preventing land use fragmentation, which will not only lead to ecological damage, but also increase carbon emissions.

## Figures and Tables

**Table 1:** List of data used in this study.

No.	Data type	Description	Source
1	Carbon emission	Inventory of carbon emission data for 2000/2005/2010/2015/2017, 5 periods	https://www.ceads.net/user/index.php?id=1057&lang=en (accessed on 5 April 2022)
2	Population	Population data for 2000/2005/2010/2015/2017, 5 periods	The statistical yearbooks of Sichuan Province and Chongqing Municipality
3	Economy	Gross Domestic Product (GDP) data for 2000/2005/2010/2015/2017, 5 periods	The statistical yearbooks of Sichuan Province and Chongqing Municipality
4	Consumption	Total Retail Sales of Consumer Goods data for 2000/2005/2010/2015/2017, 5 periods	The statistical yearbooks of Sichuan Province and Chongqing Municipality
5	Land use	Land use raster data of Sichuan basin for 2000/2005/2010/2015/2018, 5 periods	https://www.resdc.cn/data.aspx?DATAID=184 (accessed on 5 April 2022)
6	Administrative area	143 county-boundary vector data	https://www.resdc.cn/data.aspx?DATAID=202 (accessed on 5 April 2022)

**Table 2:** Descriptive of variables.

Variables		Definition
Dependent variable	C	Carbon emissions
Independent variable	E	Land-use structure information entropy
Control variables	POP	Population
	GDP	Gross Domestic Product
	RSC	Total Retail Sales of Consumer Goods

**Table 3:** Regression results of spatial Durbin model.

Index	Variables	Coef.	*z*	*p*-Value
Main	E	8.20	10.74	0.000
	POP	1.40	6.32	0.000
	GDP	0.37	2.82	0.005
	RSC	0.45	4.22	0.000
Wx	E	−3.38	−1.85	0.064
	POP	0.19	0.50	0.616
	GDP	0.07	0.24	0.809
	RSC	−1.08	−4.79	0.000
Spatial rho		0.53	12.82	0.000

Notes: R^2^ = 0.18, log-likelihood = −424.87.

**Table 4:** Decomposition of spillover effects.

Index	Variables	Coef.	*z*	*p*-Value
Direct effect	E	8.35	9.65	0.000
	POP	1.51	7.12	0.000
	GDP	0.42	3.16	0.002
	RSC	0.34	0.10	0.001
Indirect effect	E	2.03	0.57	0.570
	POP	1.92	2.93	0.003
	GDP	0.54	0.91	0.362
	RSC	−1.73	−4.04	0.000
Total effect	E	10.38	2.56	0.011
	POP	3.43	4.95	0.000
	GDP	0.96	1.49	0.136
	RSC	−1.39	0.47	0.003

Notes: R^2^ = 0.18, log-likelihood= −424.87.

## Data Availability

The data presented in this study are available on request from the corresponding author. The data are not publicly available due to privacy.
